# Machine learning identifies novel coagulation genes as diagnostic and immunological biomarkers in ischemic stroke

**DOI:** 10.18632/aging.205706

**Published:** 2024-04-03

**Authors:** Jinzhi Liu, Zhihua Si, Ju Liu, Xu Zhang, Cong Xie, Wei Zhao, Aihua Wang, Zhangyong Xia

**Affiliations:** 1 Department of Gerontology, The First Affiliated Hospital of Shandong First Medical University and Shandong Provincial Qianfoshan Hospital, Jinan, Shandong Province, China; 2 Department of Neurology, Liaocheng People’s Hospital and Liaocheng Clinical School of Shandong First Medical University, Liaocheng, Shandong Province, China; 3 Department of Gerontology, Shandong Provincial Qianfoshan Hospital, Cheeloo College of Medicine, Shandong University, Jinan, Shandong Province, China; 4 Department of Geriatric Neurology, The First Affiliated Hospital of Shandong First Medical University and Shandong Provincial Qianfoshan Hospital, Jinan, Shandong Province, China; 5 Department of Neurology, The First Affiliated Hospital of Shandong First Medical University and Shandong Provincial Qianfoshan Hospital, Shandong Institute of Neuroimmunology, Shandong Key Laboratory of Rheumatic Disease and Translational Medicine, Jinan, Shandong Province, China; 6 Laboratory of Microvascular Medicine, Medical Research Center, Shandong Provincial Qianfoshan Hospital, Cheeloo College of Medicine, Jinan, Shandong Province, China; 7 Department of Neurology, Liaocheng People’s Hospital, Cheeloo College of Medicine, Liaocheng, Shandong Province, China

**Keywords:** ischemic stroke, coagulation genes, immune infiltration analysis, machine learning, diagnosis

## Abstract

Background: Coagulation system is currently known associated with the development of ischemic stroke (IS). Thus, the current study is designed to identify diagnostic value of coagulation genes (CGs) in IS and to explore their role in the immune microenvironment of IS.

Methods: Aberrant expressed CGs in IS were input into unsupervised consensus clustering to classify IS subtypes. Meanwhile, key CGs involved in IS were further selected by weighted gene co-expression network analysis (WGCNA) and machine learning methods, including random forest (RF), support vector machine (SVM), generalized linear model (GLM) and extreme-gradient boosting (XGB). The diagnostic performance of key CGs were evaluated by receiver operating characteristic (ROC) curves. At last, quantitative PCR (qPCR) was performed to validate the expressions of key CGs in IS.

Results: IS patients were classified into two subtypes with different immune microenvironments by aberrant expressed CGs. Further WGCNA, machine learning methods and ROC curves identified ACTN1, F5, TLN1, JMJD1C and WAS as potential diagnostic biomarkers of IS. In addition, their expressions were significantly correlated with macrophages, neutrophils and/or T cells. GSEA also revealed that those biomarkers may regulate IS via immune and inflammation. Moreover, qPCR verified the expressions of ACTN1, F5 and JMJD1C in IS.

Conclusions: The current study identified ACTN1, F5 and JMJD1C as novel coagulation-related biomarkers associated with IS immune microenvironment, which enriches our knowledge of coagulation-mediated pathogenesis of IS and sheds light on next-step *in vivo* and *in vitro* experiments to elucidate the relevant molecular mechanisms.

## INTRODUCTION

The mortality and disability rate of patients with ischemic stroke (IS) is high, thus greatly threatening the life of IS patients [1]. Early diagnosis, treatment and actively controlling the complications of IS patients are the basis for improving their prognosis [2]. However, it remains unclear what causes high incidence of IS. In addition to the traditional risk factors such as hypertension, heart disease, diabetes and hyperlipidemia [3–5], it is believed that IS a polygenic complex disease from the genetic perspective, and genetic factors play an important role in the pathogenesis of IS [6, 7].

Coagulation is a complex process mediated by multiple genes and pathways [8]. In recent years, with the deepening understanding of the physiological and pathological functions of the members of the coagulation system, it has been found that the members of the coagulation system play an important role in the pathogenesis of IS [9–11]. Moreover, studies have shown that polymorphisms of coagulation genes (CGs) are associated with the risk of IS. For example, Casas et al. found that a mutation on prothrombin (20210G>A) was positively associated with IS [12]. Xu et al. found in Han Chinese population that -455A/G mutation on beta-fibrinogen was correlated with the risk of IS [13]. In addition, multiple coagulation factors, such as serotonin [14], GPIb and GPVI [15], are involved in both thrombosis and inflammation, suggesting that there is a crosstalk between coagulation and inflammation, which may contribute to the occurrence of IS via immunothrombosis. Therefore, a comprehensive and systematic study on the role of CGs in IS will strengthen our understanding of the coagulation-related genetic background of IS and further provide novel theoretical basis for the treatment of IS.

Machine learning has become a powerful tool for disease diagnosis, classification, and prediction. Generally, machine learning is classified into deep, unsupervised and supervised learning based on different purposes [16]. In the context of supervised learning, different algorithms have been developed, such as random forest (RF), support vector machine (SVM), generalized linear model (GLM) and extreme-gradient boosting (XGB). Each method has its own strength and limitations as reviewed by Jiang et al. [17] and Uddin et al. [18]. Thus, using different machine learning methods to analyze big data could help us identify key diagnostic biomarkers.

To achieve above purpose, we utilized public sequencing data for mining key CGs in IS via weighted gene co-expression network analysis (WGCNA) and machine learning, and analyzed the relationship between key CGs and immune environment of IS by single sample gene set enrichment analysis (ssGSEA), consensus clustering and correlation analyses. In addition, we explored the potential molecular mechanisms of key CGs in regulating IS by GSEA. Furthermore, the diagnostic potential of key CGs was explored and a coagulation-related diagnostic nomogram was established in IS. The pipeline and key findings of the current study were displayed in Supplementary Figure 1. We hope our study could facilitate the diagnosis, treatment and further unveiling the pathogenesis of IS.

## RESULTS

### Ten differentially expressed CGs (DECGs) were identified between IS and healthy controls (HC)

**Figure 1:**
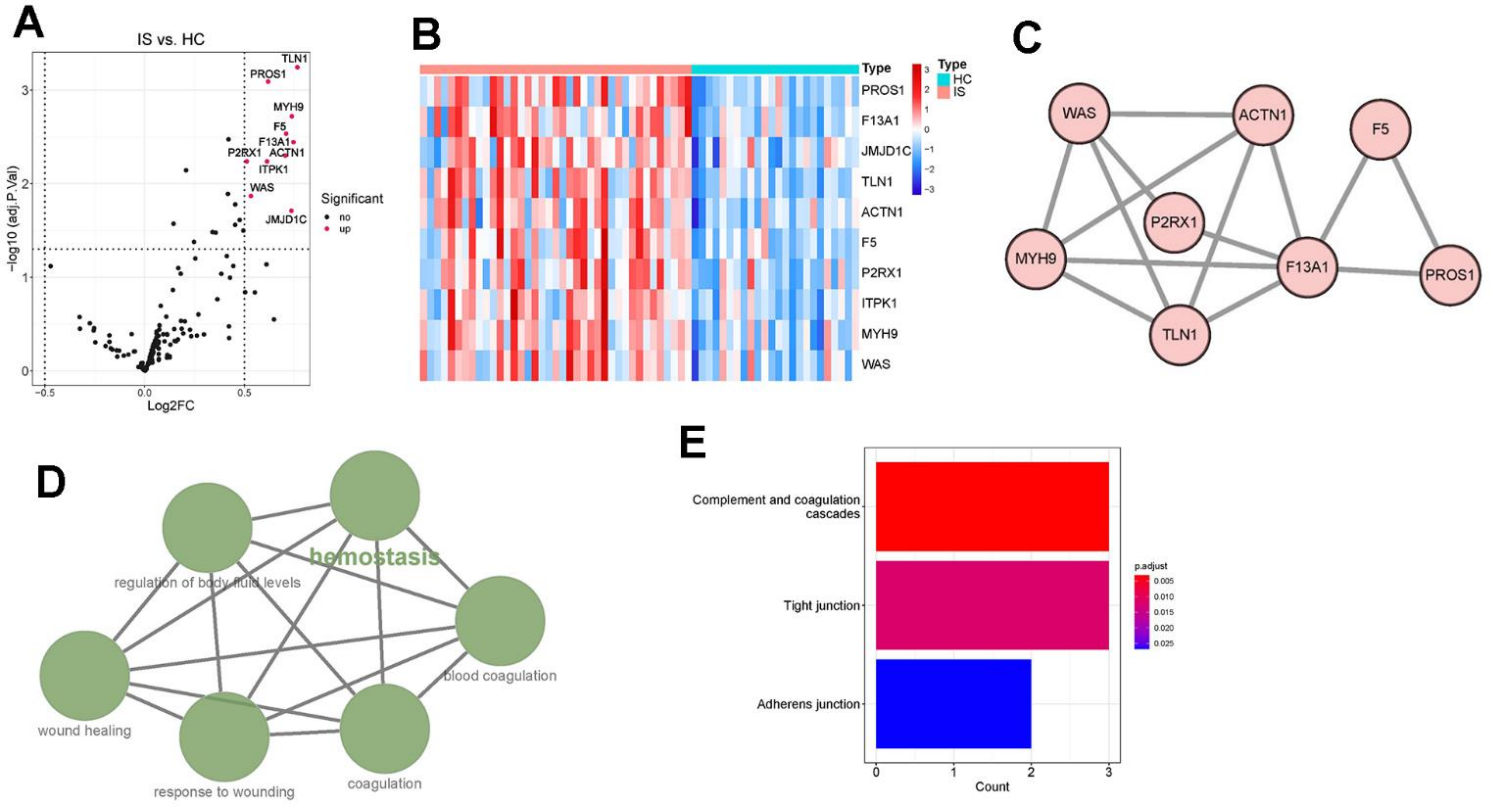
**Identification and functional enrichment analysis of differentially expressed coagulation genes (DECGs).** (**A**) Volcano plot demonstrating an overview of DECGs, in which red dots indicate upregulated CGs in IS. (**B**) Heatmap showing the increased or decreased expressions of DECGs with the hierarchical clustering for ischemic stroke (IS) and healthy control (HC) groups. The colored column sidebar at the top indicates the type of samples (coral- IS; cyan-HC). (**C**) Visualization for the predicted results of protein-protein interaction (PPI) network among DECGs via STRING and Cytoscape. Each node represents a protein, and each line refers an interaction. Line thickness indicates the strength of interaction. (**D**) A functionally grouped network of enriched GO terms and pathways was generated for DECGs by ClueGO. GO terms and pathways are represented as nodes, and the node size is proportional to the enrichment significance. The most significant term (hemostasis) is considered to be the leading term and it is highlighted in the network. (**E**) Bar chart showing that DECGs were significantly enriched in KEGG pathways of complement and coagulation cascades, tight junction and adherens junction.

By limma, ten DECGs, including talin 1 (TLN1), protein S (PROS1), myosin heavy chain 9 (MYH9), coagulation factor V (F5), coagulation Factor XIII A Chain (F13A1), actinin alpha 1 (ACTN1), purinergic receptor P2X 1 (P2RX1), inositol-tetrakisphosphate 1-kinase (ITPK1), WASP actin nucleation promoting factor (WAS) and Jumonji domain containing 1C (JMJD1C), were found to be significantly upregulated in IS group compared with those in HC group (Figure 1A, 1B). In the predicted PPI network, we observed that TLN1, PROS1, MYH9, F5, F13A1, ACTN1, P2RX1 and WAS had direct and/ or indirect interactions with each other (Figure 1C). ClueGO analysis revealed that the DECGs were clustered into six interacted functional groups, including hemostasis, regulation of body fluid levels, blood coagulation, wound healing, response to wounding and coagulation (Figure 1D). Further functional analysis on those DECGs showed that they were remarkably enriched in the pathways of complement and coagulation cascades, tight junction and adherent junction (Figure 1E). In consideration that all those functional groups and pathways are closely associated with immune and neuroinflammation [19–21], we next investigated whether there was crosstalk between DECGs and immune/ inflammation of IS.

### DECGs had close relationship with immune environment of IS

**Figure 2:**
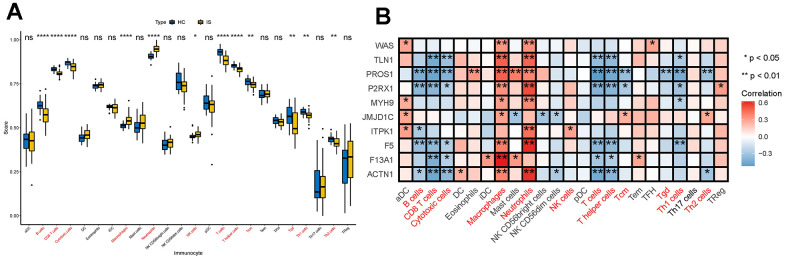
**Interactions between DECGs and immune cells.** (**A**) Box plot showing the difference of the proportions of 28 immune cells between IS and HC samples. In the x-axis, the significant differentially infiltrated immune cells were marked in red color. The value shown in the y-axis was the enrichment score of immune cells calculated by ssGSEA. ^*^p <0.05, ^**^p <0.01, ^****^p <0.0001, ^ns^p > 0.05. (**B**) Heatmap demonstrating the correlation results between the expressions of DECGs and 28 immune cells. The immune cells in red color at the bottom are significant differentially infiltrated immune cells between IS and HC. The color of each tiny box indicates if they are positive-correlated (red) or negative-correlated (blue). ^*^p <0.05, ^**^p <0.01.

Immune cells are the main players in the immune and inflammatory system. Therefore, we first explored whether the infiltration of immune cells between IS and HC were different by calculating the proportions of infiltrating immune cells in IS and HC samples by ssGSEA (Supplementary Table 1). By comparison, we found significantly lower levels of B cells, CD8 T cells, cytotoxic cells, T cells, helper T cells, Tcm, Tgd, Th1 cells and Th2 cells and higher levels of macrophages, neutrophils and NK cells in IS group (Figure 2A), indicating that immune environment is altered in IS. Further Spearman analysis partially supported our hypothesis that DECGs had a close relationship with the immune microenvironment of IS. In detail, PROS1, P2RX1, ITPK1, F5 and ACTN1 were significantly negatively correlated with B cells. TLN1, PROS1, P2RX1, F5, F13A1 and ACTN1 had remarkably negative associations with CD8 T cells and cytotoxic cells. Except ITPK1, the other 9 DECGs were positively correlated with macrophages (p <0.05). As for neutrophils, they have positive relationship with DECGs except JMJD1C and F13A1 (p <0.01). NK cells were only positively correlated with ITPK1 (p <0.05). Also, we observed that TLN1, PROS1, P2RX1, F5, F13A1 and ACTN1 had significantly negative correlations with T cells and T helper cells. In addition, PROS1, P2RX1 and JMJDC1 were remarkably correlated with Tcm. Tgd was only significantly related to the expression of PROS1. Th1 cells had significant correlations with TLN1, PROS1, P2RX1, MYH9 and F5, while Th2 cells were remarkably correlated with PROS1, JMJD1C and ACTN1 (Figure 2B). Collectively, DECGs may be involved in IS immune microenvironment by interacting with common and/ or specific immune cells.

### IS patients were divided into two clusters with different characteristics based on DECGs

**Figure 3:**
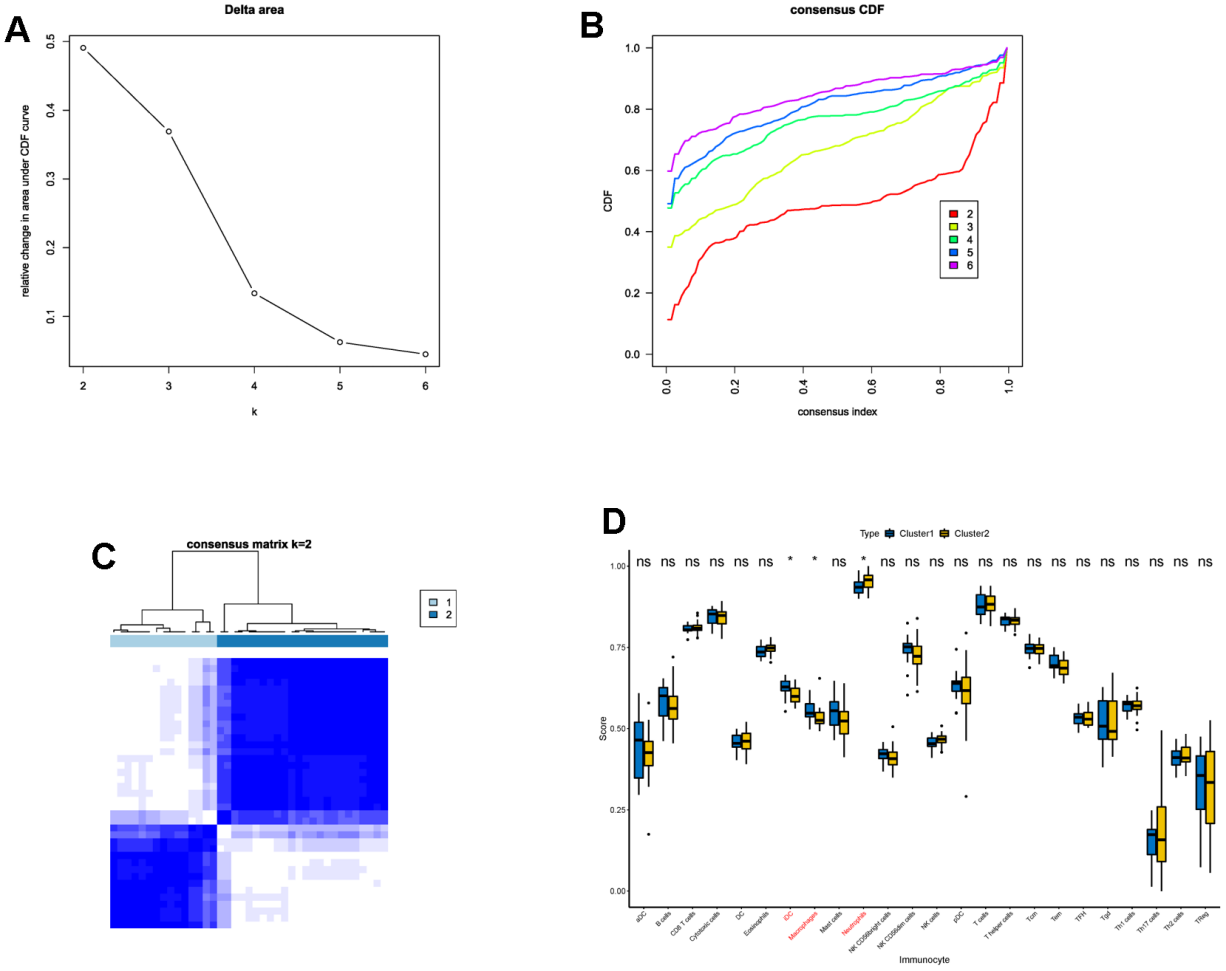
**Consensus clustering of IS patients based on DECGs.** (**A**) Delta area plot displaying the relative change in area under CDF curve between K and K-1. (**B**) Cumulative distribution function (CDF) plot showing consensus clustering under K = 2, 3, 4, 5, 6, and when K = 2, the classification is stable. (**C**) Consensus matrix at K = 2. The values of the consensus matrix are shown in white to dark blue from 0 (impossible to be clustered together) to 1 (always clustered together), and the consensus matrix was arranged according to the consensus clustering (dendrogram above the heatmap). The bars between the dendrogram and heatmap represent the molecular subtypes. All results in A-C indicate that the sample clustering was stable and robust that the boundary of the consensus matrix was clear. (**D**) Box plot showing the difference of the proportions of 28 immune cells between cluster 1 and cluster 2. In the x-axis, the significant differentially infiltrated immune cells were marked in red color. The value shown in the y-axis was the enrichment score of immune cells calculated by ssGSEA. ^*^p <0.05, ^ns^p > 0.05.

**Figure 4:**
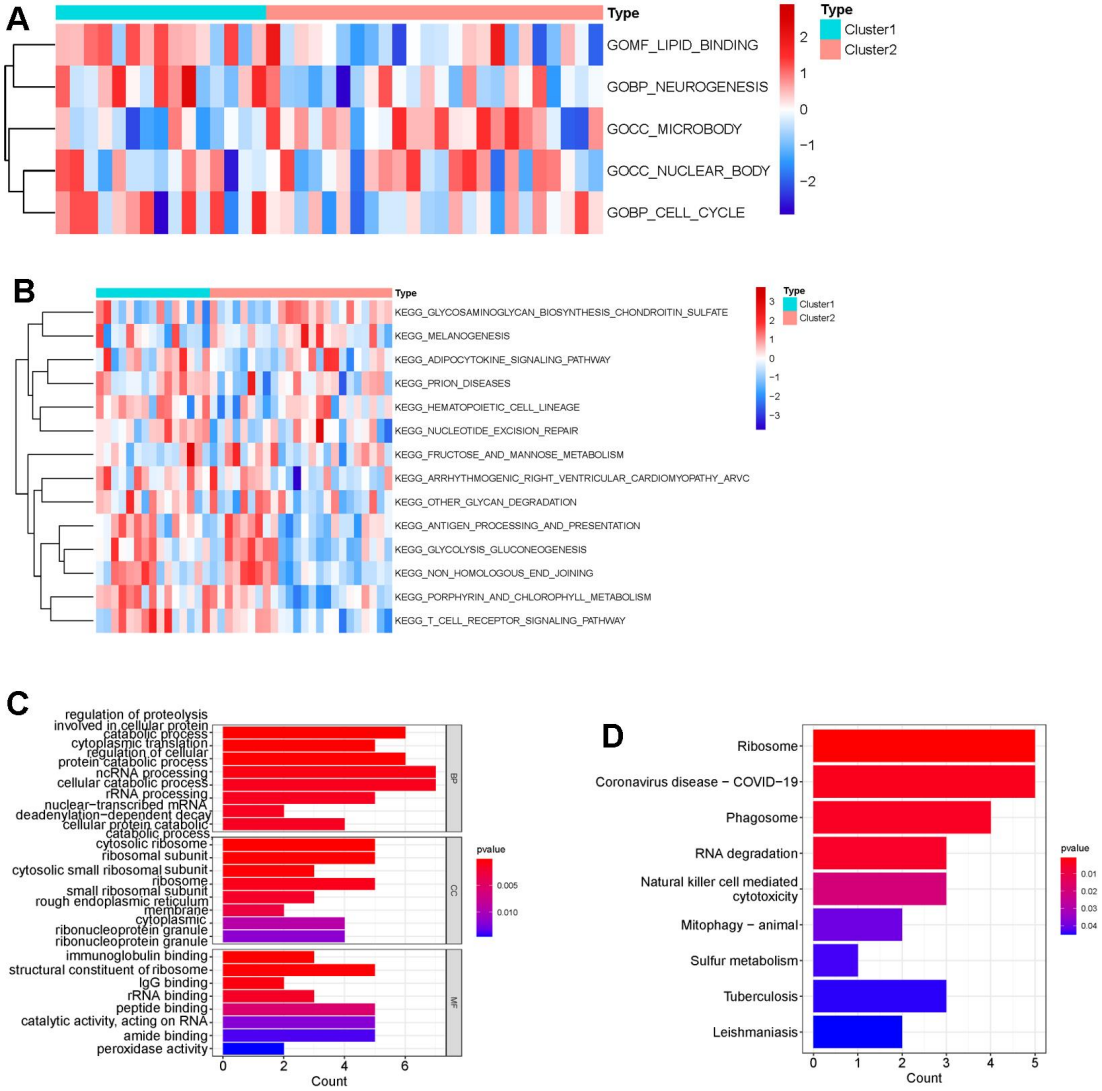
**Difference of functional enrichment of genes in cluster 1 and cluster 2.** Heatmap displaying differentially enriched GO terms (**A**) and KEGG pathways (**B**) between cluster 1 and cluster 2 by calculating enrichment score using GSVA. The tiny boxes in the heatmap are colored by mean GSVA scores from dark red to dark blue. (**C**) Bar chart displaying GO terms sorted by adjusted p value in the category of biological process (BP), cellular component (CC) and molecular function (MF) that DEGs between cluster 1 and cluster 2 are enriched in. (**D**) Bar chart showing that DEGs between cluster 1 and cluster 2 were significantly enriched in nine KEGG pathways using p <0.05 as cutoff.

Next, we performed consensus clustering, and found that those DECGs could divide IS patients into two subgroups (cluster 1 and cluster 2, Figure 3A–3C). Furthermore, we analyzed and compared the characteristics of two clusters. We observed that cluster 1 had less neutrophils and more iDC and macrophages compared to cluster 2 (p <0.05, Figure 3D). In addition, gene set variation analysis (GSVA) results revealed that physiological function related to GO terms of lipid binding, neurogenesis, microbody, nuclear body, cell cycle (Figure 4A), and pathways associated with metabolism, cell behavior and immune system, such as adipocytokine pathway, glycosaminoglycan biosynthesis, nucleotide excision repair, antigen processing and presentation, glycolysis/gluconeogenesis, non-homogenous end joining and T cell receptor signaling pathway (Figure 4B) were differentially enriched between two clusters, indicating that IS patients in two clusters may vary in metabolism, cell behavior and immune. Similar to GSVA results, biological function analysis showed that DEGs between two clusters (Supplementary Table 2) were significantly enriched in the BPs of cellular catabolic process, ncRNA processing, cytoplasmic translation, CCs related to ribosome, endoplasmic reticulum membrane, MFs of immunoglobulin binding, structural constituent of ribosome and IgG binding (Figure 4C) and pathways of ribosome, COVID-19, RNA degradation and phagosome (Figure 4D), which also had close relationship with metabolism, cell behavior and immune.

### Five key CGs were identified in IS by WGCNA and machine learning

**Figure 5:**
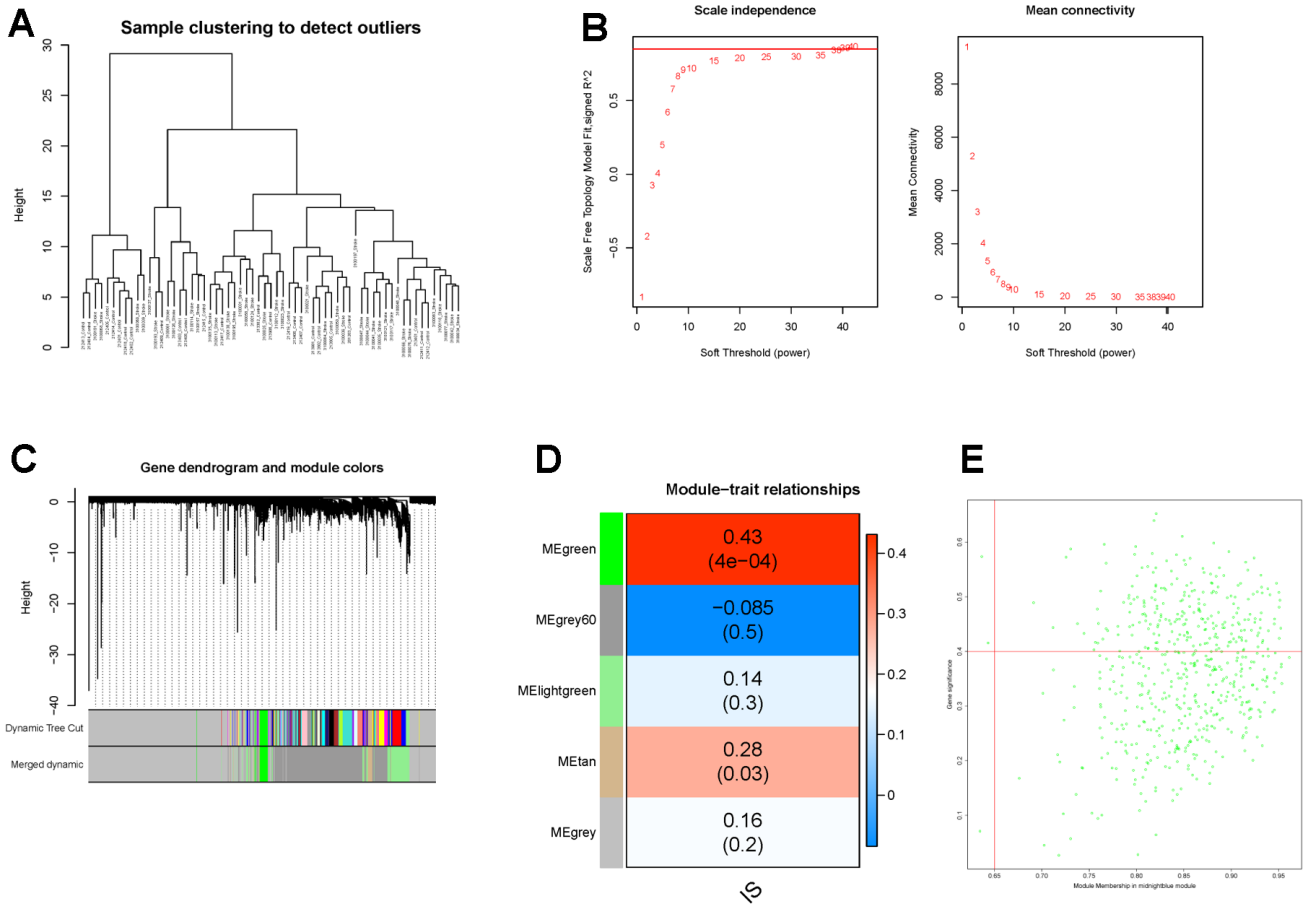
**Identification of IS-related genes by WGCNA.** (**A**) The clustering dendrogram of samples shows that samples were well divided into two groups with no outlier. (**B**) Determination of the soft-threshold to achieve the scale-free network. The left panel shows the influence of soft-threshold power on the scale-free fit index, and the right panel shows the impact of soft-threshold power on the mean connectivity. (**C**) Gene dendrogram obtained by hierarchical clustering and modules with different colors assigned by dynamic tree cutting. (**D**) Heatmap displaying the correlation between modules and IS. Each row correlates to a module eigengene, and each cell includes the corresponding correlation and p-value. (**E**) The scatterplot of gene significance (GS) versus module membership (MM) in the green module.

**Figure 6:**
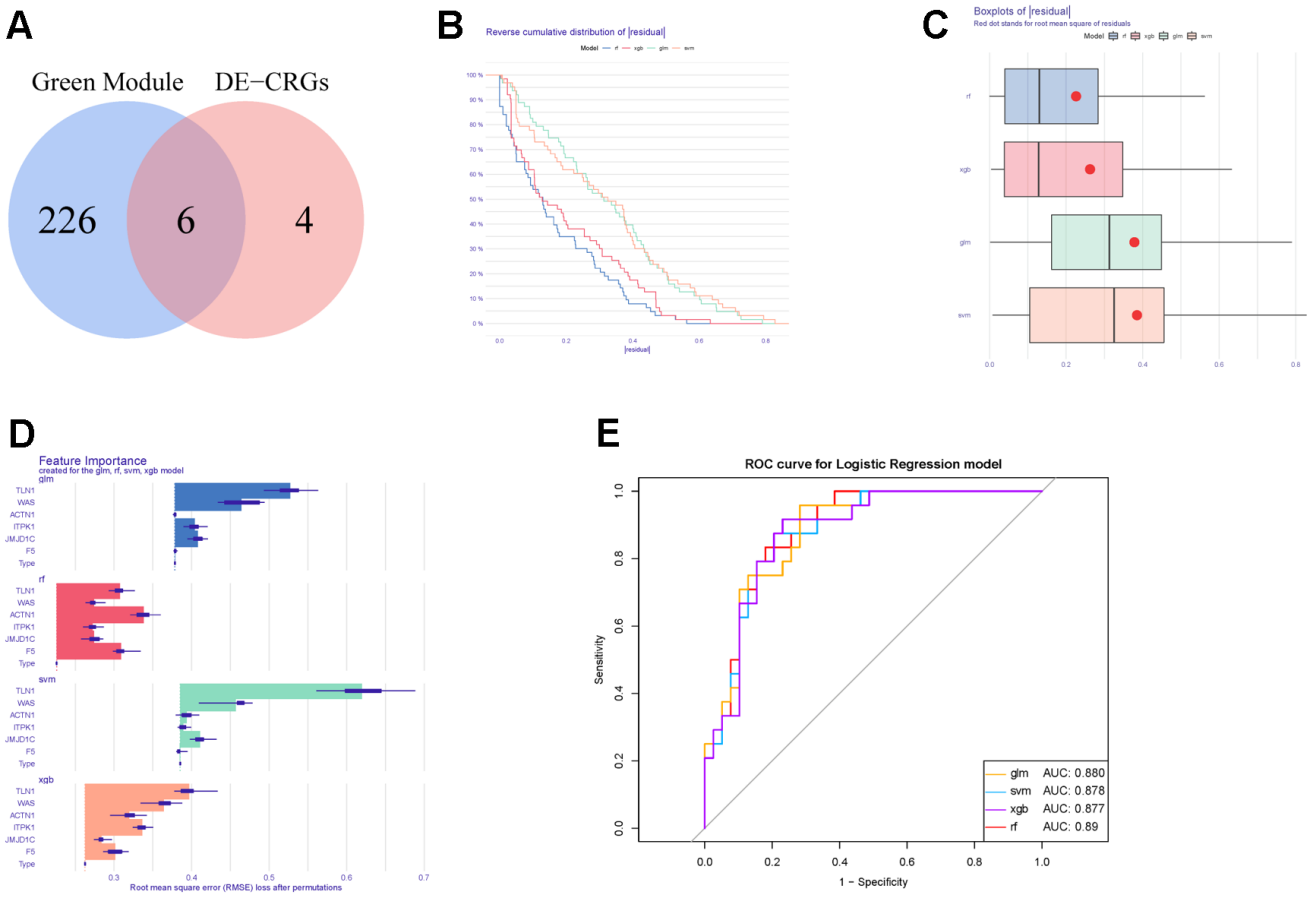
**Construction and evaluation of different machine learning models.** (**A**) Venn diagram showing the intersection between DECGs and WGCNA modular genes. (**B**) Reverse cumulative distribution of residuals in four machine learning models. (**C**) Boxplots of residuals in four machine learning methods. (**D**) The feature importance of CGs in each machine learning model. (**E**) ROC curves of four machine learning models for IS diagnosis.

Thereafter, WGCNA and machine learning were carried out to identify key CGs involved in IS. Firstly, we performed WGCNA to detect DECGs associated with IS. Sample clustering result showed that there was no outlier in the dataset (Figure 5A). Then according to WGCNA package of R, we selected 38 as the optimal soft-threshold to construct the scale-free network (Figure 5B). Subsequently, a total of 20 modules were divided by dynamic tree cutting, and 5 modules (green, grey60, light green, tan and grey) were finally obtained after modular merging (Figure 5C). Among the five modules, the green module had the strongest positive correlation with IS (R^2^ = 0.43, p-value < 0.001) (Figure 5D). Then genes in the green module were further filtered by |gene significance (GS)| > 0.4 and |module membership (MM)| > 0.65, and a total of 232 genes were selected and defined as IS-related genes (Figure 5E). Thus, six candidate genes (TLN1, F5, ACTN1, ITPK1, WAS and JMJD1C) were obtained by intersecting DECGs with WGCNA modular genes (Figure 6A).

**Figure 7:**
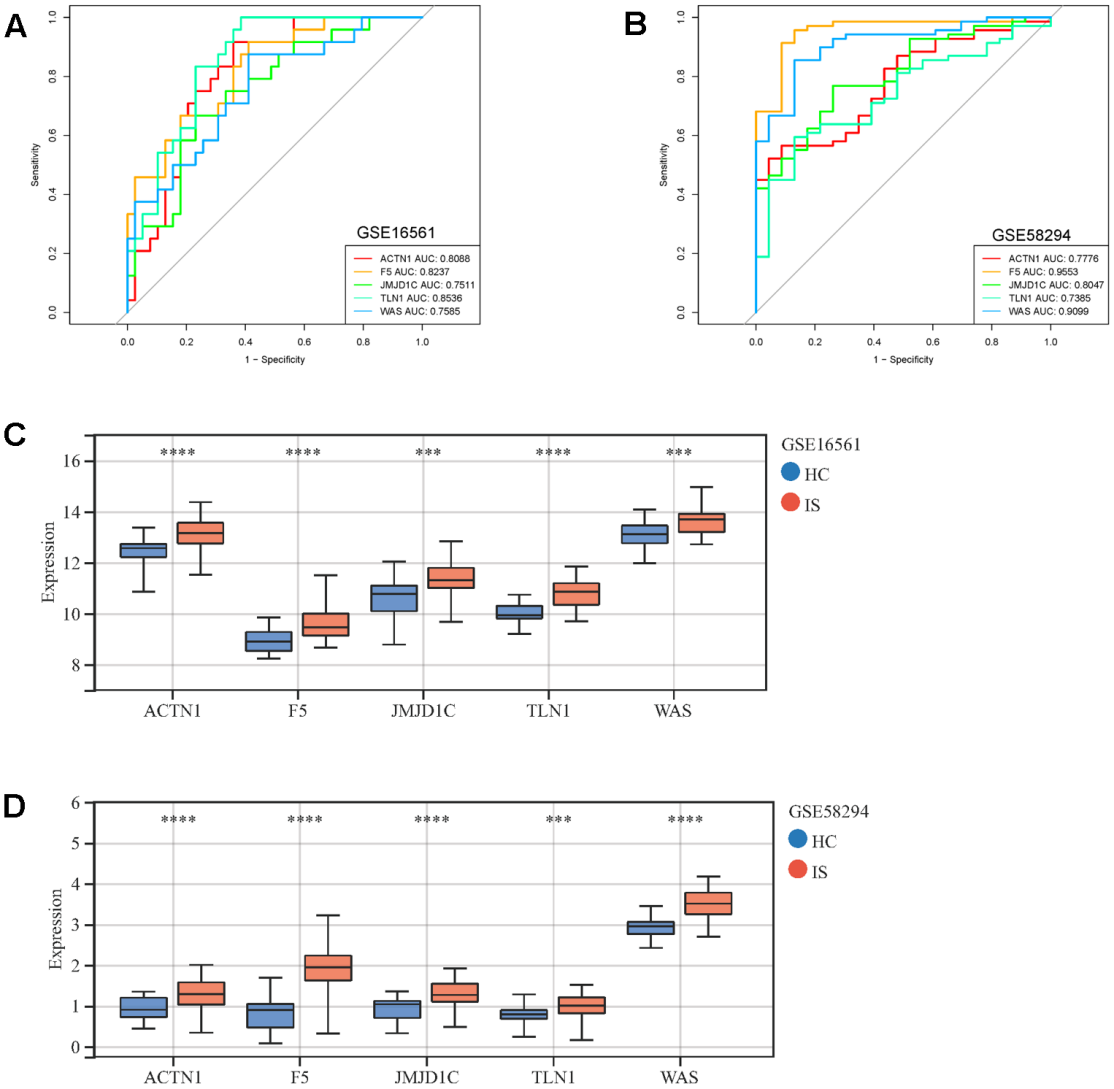
**ROC curves and expressions of key CGs in IS.** ROC curves display the performance (sensitivity and specificity) of key CGs in IS diagnosis in GSE16561 (**A**) and GSE58294 (**B**). Comparison of key CRGs’ expressions between IS and HC samples in GSE16561 (**C**) and GSE58294 (**D**). ^***^p <0.001, ^****^p <0.0001.

**Figure 8:**
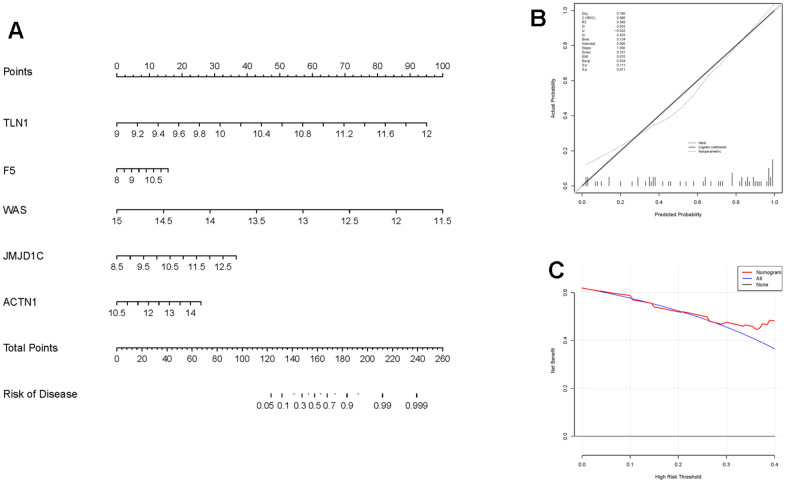
**Construction and evaluation of the nomogram in IS.** (**A**) The nomogram constructed based on key CGs for IS diagnosis. The line segment corresponding to each variable is marker with a scale, which represents the possible value range of the variable, and the length of the line segment reflects the contribution of the variable to the outcome event. The value of each variable was given a score on the point scale axis. (**B**) Calibration curve displaying the accuracy of the nomogram. The x-axis represents the predicted IS risk. The y-axis represents the actual diagnosed IS. The solid line represents a perfect prediction by an ideal model. The dot line represents the performance of the nomogram, of which a closer fit to the solid line represents a better prediction. (**C**) The decision curve showing the clinical utility of the nomogram.

Moreover, those six candidate genes were input into RF, SVM, GLM and XGB algorithms to evaluate their importance. We found that RF algorithm was the most accurate one with the lowest residual value (Figure 6B, 6C), in which the rank order of feature importance was ACTN1, F5, TLN1, JMJD1C, WAS and ITPK1 (Figure 6D). Moreover, RF model had the best performance to distinguish IS from HC samples with the AUC of 0.89 (Figure 6E). Furthermore, we investigated the diagnostic potential of ACTN1, F5, TLN1, JMJD1C, WAS and ITPK1 in IS by receiver operating characteristic (ROC) curves, which showed that TLN1, F5, ACTN1, WAS and JMJD1C may be potential diagnostic biomarkers in both testing (GSE16561, Figure 7A) and validation (GSE58294, Figure 7B) cohorts. Therefore, TLN1, F5, ACTN1, WAS and JMJD1C were considered as key CGs in IS. We found that the expression patterns of TLN1, F5, ACTN1, WAS and JMJD1C were consistent in the testing and validation cohorts (Figure 7C, 7D), further suggesting their importance in IS. At last, TLN1, F5, ACTN1, WAS and JMJD1C were used to construct the nomogram to predict the risk of IS onset (Figure 8A). Both calibration curve (Figure 8B) and decision curve (Figure 8C) demonstrated the good performance of nomogram to predict IS.

### GSEA revealed the potential mechanisms of key CGs in regulating IS

**Figure 9:**
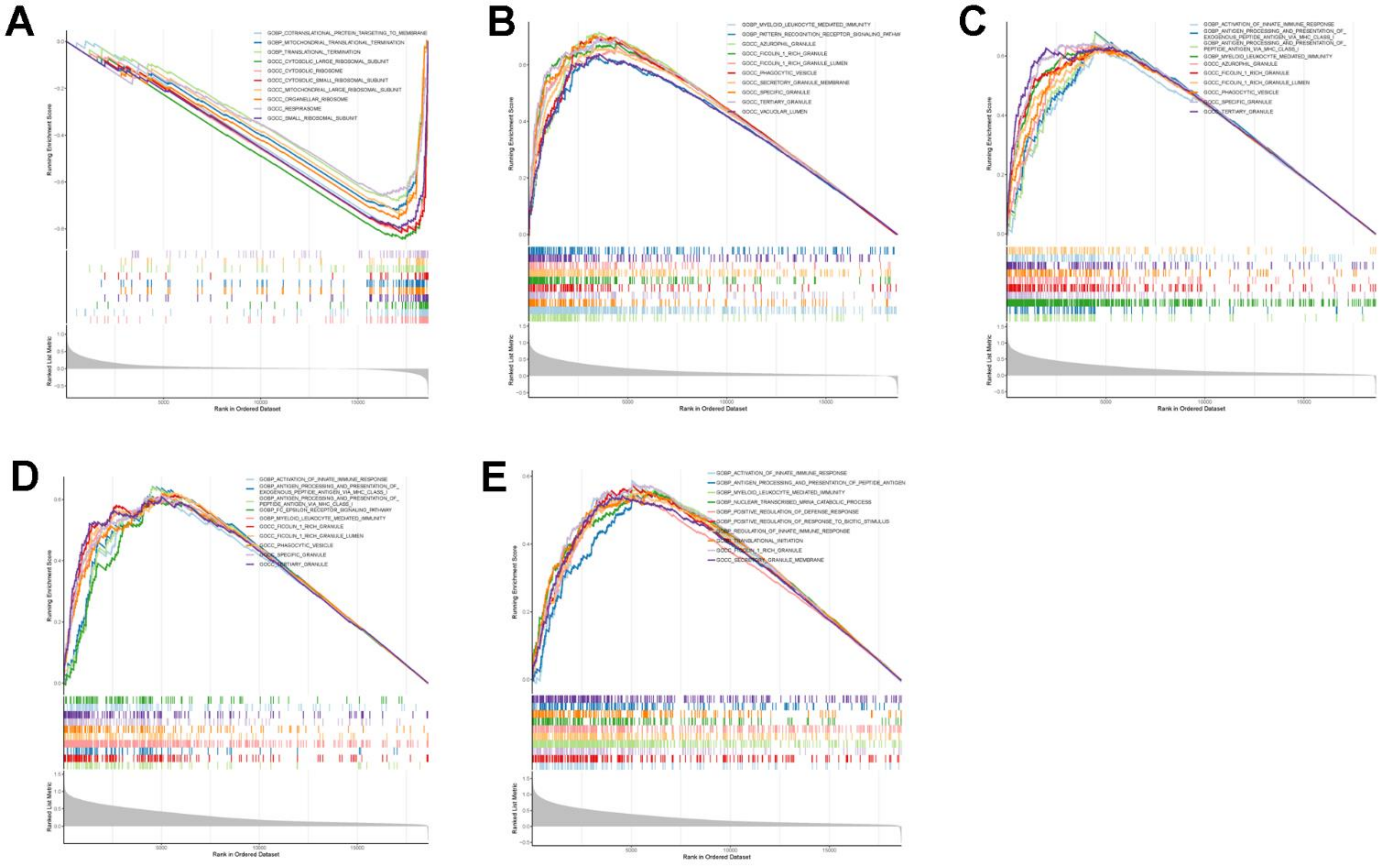
**GSEA enrichment analysis on each key CGs based on “GO term” reference gene sets.** GSEA plot was based on the gene expression profiles of key CG-high expression group compared with key CG-low expression group. The vertical colorful lines represent the running enrichment scores as the projection of individual genes onto the horizontal ranked gene list. The bottom ranking matric in gray, moving from above zero (positively correlated) to below zero (negatively correlated), measures a gene’s correlation with the phenotype profile. (**A**) Actinin alpha 1 (ACTN1). (**B**) Talin 1 (TLN1). (**C**) Coagulation factor V (F5). (**D**) WASP actin nucleation promoting factor (WAS). (**E**) Jumonji domain containing 1C (JMJD1C).

**Figure 10:**
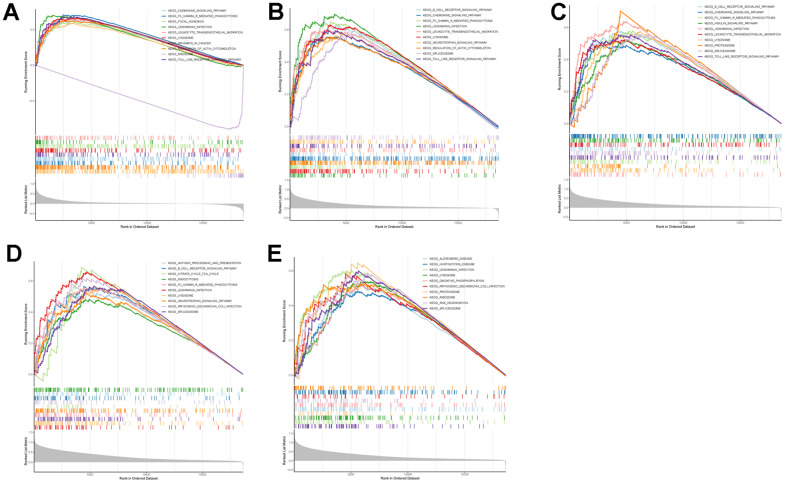
**GSEA enrichment analysis on each key CGs based on “KEGG pathway” reference gene sets.** GSEA plot was based on the gene expression profiles of key CG-high expression group compared with key CG-low expression group. The vertical colorful lines represent the running enrichment scores as the projection of individual genes onto the horizontal ranked gene list. The bottom ranking matric in gray, moving from above zero (positively correlated) to below zero (negatively correlated), measures a gene’s correlation with the phenotype profile. (**A**) Actinin alpha 1 (ACTN1). (**B**) Talin 1 (TLN1). (**C**) Coagulation factor V (F5). (**D**) WASP actin nucleation promoting factor (WAS). (**E**) Jumonji domain containing 1C (JMJD1C).

To further explore the potential mechanisms of TLN1, F5, ACTN1, WAS and JMJD1C in regulating IS, we performed GSEA analysis. As for GO terms, ACTN1 was more related to protein synthesis and processing, such as translation termination and cytosolic ribosome (Figure 9A), while TLN1, F5, WAS and JMJD1C were more involved in immune-related function, such as myeloid leukocyte mediated immunity, activation of innate immune response, antigen processing and presentation (Figure 9B–9E). As for KEGG pathways, ACTN1, TLN1, F5 and WAS were mainly enriched in immune-related pathways, such as chemokine signaling pathway, B cell receptor signaling pathway, Fc gamma R-mediated phagocytosis, antigen processing and presentation (Figure 10A–10D), while JMJD1C was more likely participated in pathways related to neuroinflammation, such as Alzheimer’s disease, Huntington’s disease and oxidative phosphorylation (Figure 10E). Taken together, those findings suggest that TLN1, F5, ACTN1, WAS and JMJD1C may regulate the development of IS mainly by immune and inflammation.

### The expression patterns of ACTN1, F5 and JMJD1C were validated *in vivo*

**Figure 11:**
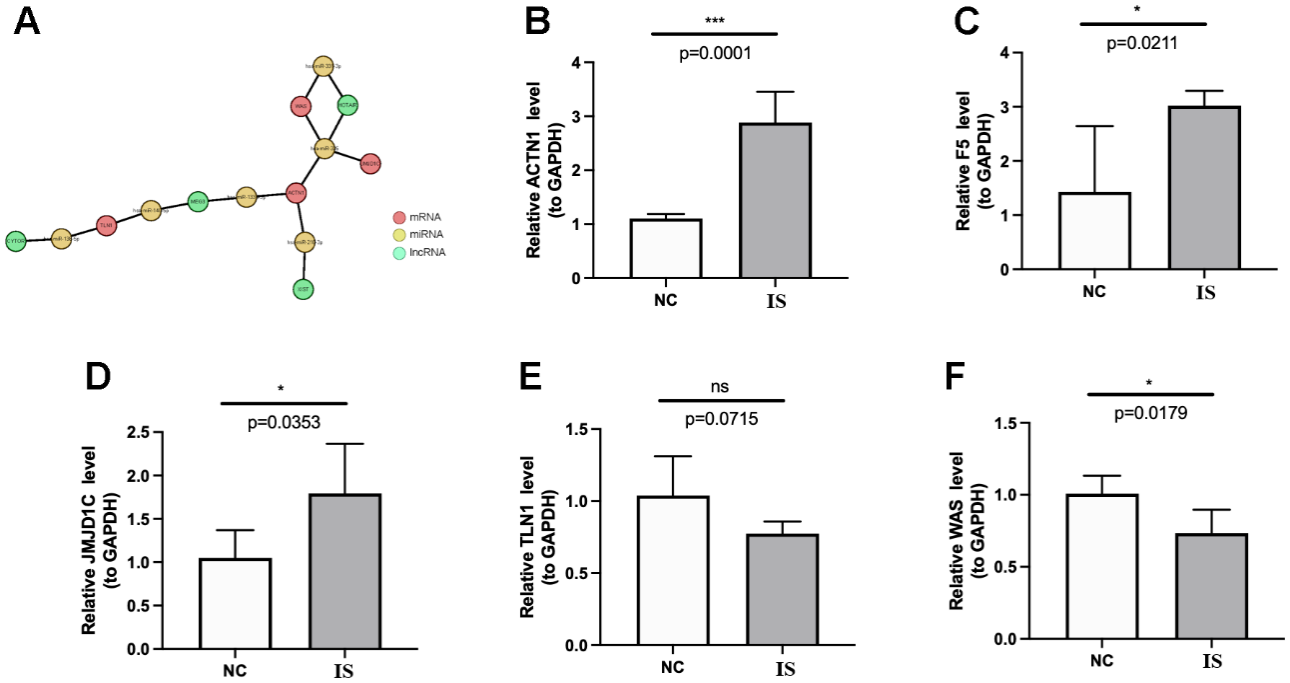
**Regulatory network and qPCR validation.** (**A**) The lncRNA-miRNA-key CGs regulatory network. The coral, green and yellow circle represents mRNA, lncRNA and miRNA, respectively. The lines between circles mean that there are predicted regulatory pairs. The expressions of ACTN1 (**B**), F5 (**C**), JMJD1C (**D**), TLN1 (**E**) and WAS (**F**) were detected and compared between IS and HC samples by qPCR. The results were shown as mean ± S.D. ^*^p <0.05, ^***^p <0.001, ^ns^p > 0.05.

Finally, a mRNA-miRNA-lncRNA network was constructed, which was composed of 4 key genes (TLN1, ACTN1, WAS and JMJD1C), 6 miRNAs (mir-138-5p, mir-140-5p, mir-133a-3p, mir-210-3p, mir-326, mir-331-3p) and 4 lncRNAs (CYTOR, MEG3, HOTAIR, XIST) (Figure 11A). In the mRNA-miRNA-lncRNA network, multiple regulatory relationships were predicted, for instance, MEG3 and HOTAIR may regulate the expression of ACTN1 by interacting with hsa-miR-133a-3p and hsa-miR-326, respectively. Moreover, we validated the expressions of TLN1, F5, ACTN1, WAS and JMJD1C using clinical IS samples and control samples. The qPCR results showed that ACTN1, F5 and JMJD1C were significantly up-regulated in IS (Figure 11B–11D), which were consistent with sequencing results. However, the expression patterns of TLN1 and WAS conflicted with sequencing results (Figure 11E, 11F).

## DISCUSSION

Given the importance of coagulation in IS development, our current study was designed to identify key CRGs and to explore related mechanisms in IS. Based on the above goals, we first identified 10 DECRGs between IS and HC samples, which were enriched into biological functions of immune, inflammation, coagulation and wound healing. Coagulation is one of programmed phases in wound healing [22], and both coagulation and wound healing are strongly associated with inflammation and immune [19, 20, 23, 24]. Immune cells are a major part in immune system that they establish an immune microenvironment with other cells and factors. In the current study, by comparing the proportions of 28 immune cells, we found the alteration of immune microenvironment in IS. More interestingly, the expressions of 10 DECRGs were significantly correlated with immune cells, especially macrophages, neutrophils, and T cells. Macrophages can be observed in cerebral 4 days after the onset of cerebral ischemia, and after 7 days, macrophages reached the peak and then reduced, but microglia in the brain were activated and obtained the phenotype of M1 or M2 macrophages to execute pro- or anti-inflammation functions by various cytokines [25, 26]. Neutrophils appeared very early at the onset of IS [27], and neutrophils can produce MMP9, which can damage blood-brain barrier (BBB) and cause hemorrhagic transformation of IS [28]. In addition, neutrophil to lymphocyte ratio (NLR) has recently been reported as potential novel biomarkers of baseline inflammatory process and could serve as an outstanding predictor in patients with IS [29]. Invasion and infiltration by peripheral T cells play an important role in the progress of IS [30]. In the study of cerebral ischemia, the cerebral infarct area was reduced in mice devoid of T cells [31]. The importance of macrophages, neutrophils and T cells in IS has been recently reviewed by Takashi et al. [32]. Moreover, the infiltrations of macrophages and neutrophils were significantly different between two IS subtypes divided by DECGs, further demonstrating that DECGs may affect the immune microenvironment of IS via interacting with immune cells.

Next, to further select key DECRGs involved in IS, we performed WGCNA and identified six key DECRGs, including TLN1, F5, ACTN1, ITPK1, WAS and JMJD1C. By ROC curves in both training and validation sets, we found that TLN1, F5, ACTN1, WAS and JMJD1C had good performance in distinguishing IS from HC samples, indicating their ability to use as IS diagnostic biomarkers. TLN1 affects cell behaviors mediated by integrins, such as adhesion, proliferation, survival, migration and remodeling of cytoskeleton [33–35]. Those processes are also associated with the formation and stability of plaque, which contributes to the increased risk of IS. Wei et al. found that the expression of TLN1 between stable and unstable plaques from symptomatic carotid stenosis patients were significantly different [36], indicating that TLN1 may be involved in regulating plaque stability. In addition, a recent bioinformatic study also showed that TLN1 was a promising diagnostic marker correlated with neutrophils and M0 macrophages in IS [37], which was consistent with our findings. However, so far to our knowledge, the potential mechanisms of TLN1 in regulating IS remain unclarified. In our study, by GSEA method, we investigated the molecular mechanisms of TLN1 that TLN1 is involved in many immune and inflammation related pathways, such as chemokine signaling, toll-like receptor signaling and insulin signaling, which are key signaling pathways in IS development [38–40]. Multiple studies have reported that the polymorphisms of F5 were correlated with thrombosis and IS, such as c. 1691G>A [41] and c4970A>G [42]. But the study on the role of F5 aberrant expression in regulating IS remains limited. There is no report between ACTN1 and IS. ACTN1 is mainly expressed in platelets, and its variants are associated with thrombocytopenia [43, 44]. Platelets play important roles in the development of IS, including but not limited to by inducing the expressions of ICAM1, E-selectin and P-selectin [45], formation of platelet-leukocyte aggregates [46] and interacting with neutrophils [47]. Thus, it is suggested that ACTN1 may regulate IS via platelets, which need to be further explored. JMJD1C is a histone H3 lysine 9 (H3K9) demethylase, which is tightly related to lipogenesis [48] and epigenetic regulation of steroidogenesis [49] and adipogenesis [50]. One of the most important causes of cerebral ischemia is the deposition of lipids on the inner wall of blood vessels, which leads to the formation of plaque and affects blood flow. *In vitro* and *in vivo* experiments are needed to investigate whether JMJD1C regulates IS via adipogenesis or not. As for WAS, it encodes WASP and participates in acting network assembly [51], which is also associated with cell behaviors [52–54], however, how it functions in IS are not elucidated. In addition, we also try to explain the molecular mechanisms of those biomarkers by bioinformatic analysis. Interestingly, we again found that those genes were all related to immune and inflammation pathways, further supporting our hypothesis that those biomarkers may affect IS via immune and inflammation. Thus, our study identified novel coagulation marker genes and explored their potential mechanisms in IS. Those findings shed novel lights on our understanding of IS pathogenesis and may benefit the development of auxiliary diagnosis in IS and new drugs targeting those marker genes for IS treatment.

Some limitations have to be acknowledged in our study. First, our study is mainly based on public transcriptome datasets, which are relatively small sample sizes. Larger clinical cohorts should be enrolled to determine and validate the diagnostic performance and clinical utility of key CGs in future. Second, in consideration of small sample size of the dataset and the number of CGs, we used |log2FC| > 0.5 and adjusted p-value <0.05 to filter DECGs. Thus, the biological relevance and the molecular mechanisms of F5, ACTN1 and JMJD1C in regulating IS needs to be explored by *in vitro* and *in vivo* experiments. Among them, we are most interested in F5. Thus, based on above limitations, we plan to (1) collect more samples at different stages of IS to examine the expressions and locations of F5 by using qPCR and immunofluorescence; (2) treat human umbilical vein endothelial cells (HUVECs) by OGD/R method to mimic IS *in vitro*, and then overexpress F5 to see whether it affect cellular behaviors (proliferation, apoptosis, migration, angiogenesis) and expressions of IL-1β, IL-6 and TNF-α; (3) deliver F5 overexpressing lentivirus into I/R mice and detect infarct size and neuroinflammation.

In summary, for the first time, we identified novel CGs as potential IS biomarkers and investigated their relationship with immune microenvironment of IS. We hope that our findings can provide fundamental information in the pathogenesis of IS from coagulation and inflammation perspective, and support for diagnosis and treatment in IS patients in future.

## MATERIALS AND METHODS

### Date sources

All expression profiling data used in the current study were downloaded from GEO database (https://www.ncbi.nlm.nih.gov/geo/) with the following inclusion criteria: (1) more than 30 peripheral blood samples from Homo sapiens; (2) concurrent inclusion of both IS patients and non-stroke healthy controls (HC). Accordingly, two GEO datasets were obtained, including GSE16561 (IS = 39, HC = 24) from GPL6883 platform of Illumina HumanRef-8 v3.0 expression BeadChip as the training set and GSE58294 (IS = 69, HC = 23) from GPL570 platform of Affymetrix Human Genome U133 Plus 2.0 Array as an external verification set. The “oligo” R package was used to preprocess (background correction, log transformation and normalization) the raw gene expression profiles for downstream analyses. Besides, 185 human CGs were sourced from AmiGO2 database (http://amigo.geneontology.org/amigo) [55].

### Differential expression analysis and enrichment analysis

Limma (version 3.48.3) package [56] was used to obtained DECGs between IS and HC groups (|log2FC| > 0.5, adj.P.Val<0.05). Ggplot2 (version 3.3.5) and pheatmap (version 1.0.12) were performed to draw volcano and heat map, respectively. Then, GO and KEGG enrichment analysis of DECGs were performed by ClusterProfiler (version 4.4.4) [57] to obtain the function items and related pathways (p.adjust < 0.05 and count > 1). Finally, functional networks of DECGs were performed by the ClueGO plug-in of Cytoscape.

### The construction of a protein-protein interaction (PPI) network and immune infiltration analysis

The PPI network was constructed based on the STRING database (https://string-db.org/). Cytoscape was used to visualize the PPI network of DECGs. Based on 24 immune cell gene sets, the proportion of infiltrating immune cells in each sample was calculated by ssGSEA algorithm. The box plot of the proportion of infiltrating immune cells in IS and HC groups was drawn according to the calculation results, and the Wilcoxon test was used to analyze the difference of immune cell infiltration between IS and HC groups (p < 0.05). Ggplot2 (version 3.3.5) [58] was used to draw box plot. The correlation between DECGs and immune cells was calculated by Spearman analysis.

### Identification and analysis of coagulation-related subtypes

Firstly, in order to evaluate the stability of clustering and determine the optimal number of clusters, consensus clustering was performed on 39 IS samples in the data set GSE16561 based on DECGs. According to the consensus clustering results, the box plot of proportion of infiltrating immune cells in different clusters was plotted by ggplot2 package, and the Wilcoxon test was used to analyze the difference in immune cell infiltration (p < 0.05). Then, to study the genetic mechanisms involved in CG-mediated regulation, we compared DEGs between different subtypes (|log2FC| > 1 and p-value < 0.05), and GO and KEGG enrichment analyses were performed on DEGs. Finally, to further explore the pathways activated in different clusters, the msigdbr (version 7.4.1) package was used to download the GO and KEGG pathway gene sets as reference gene sets. Then, ssGSEA algorithm was performed by GSVA (version 1.40.1) package to obtain the ssGSEA score for each GO terms and pathways, and the limma package was used to compare the enrichment differences of GO terms and pathways between different subtypes.

### Screening of module genes

WGCNA (version 1.70-3) package [59] was performed to construct a co-expression network to find the gene module most related to IS. Firstly, the clustering of the IS samples was carried out to detect outlier samples to ensure the accuracy of the subsequent analysis. The soft threshold of the data was determined to achieve a scale-free topology. Based on optimal soft thresholds, we set a minimum number of genes in per module as 100 according to dynamic tree cutting algorithm. The correlation between module and IS was calculated to obtain the key module. A scatter plot was drawn for key module to show the correlation between GS and MM.

### Identification of key CGs in IS

Firstly, candidate genes were obtained by intersection of DECGs with key modular genes. Then, to further assess the importance of candidate genes in IS, 4 types of machines learning method, including RF, SVM, GLM and XGB were performed, respectively. Four models were calculated using the DALEX (version 2.3.0) package to draw the residual distribution map to obtain the best model. Next, the discriminant performance of four machine learning algorithms in GSE16561 data set was evaluated by ROC curves. Genes obtained by the algorithm with the best evaluation effect were selected as candidate key CGs for further analysis. Finally, to study the ability of candidate key CGs to distinguish between IS samples and HC samples, ROC curves of candidate key genes were drawn by pROC package [60]. The area under curve (AUC) value ≥ 0.7 was selected as diagnostic CGs. The ggplot2 (version 3.3.5) package [58] was used to show the expressions of diagnostic CGs in GSE16561 and GSE58294 data set, respectively.

### Clinical value and enrichment analysis of key CGs

Rms (version 6.1-0) package was used to construct the nomogram of key CGs, and calibration curves were drawn to assess the accuracy of the model. GSEA were performed to explore the related pathways and molecular mechanisms of key genes by ClusterProfiler (version 4.0.2) package [57] and org.Hs.eg.db package (version 3.13.0).

### Construction of ceRNA network

Firstly, miRNA targeting key CGs were predicted by miRWalk database (http://mirwalk.umm.uni-heidelberg.de). The criterion for screening miRNA was energy < -30. Then, lncRNAs interacting with selected miRNAs were predicted using the miRTarBase database (https://ngdc.cncb.ac.cn/databasecommons/database/id/167). Finally, ceRNA network was constructed based on key CGs, miRNA and lncRNA. Cytoscape was used to generate the ceRNA network.

### Experimental verification

Human peripheral blood samples were obtained from IS patients (N = 5) and healthy individuals (N = 5), who agreed to participate in the current study with written consent. This study was approved by the ethics committee of Shandong Provincial Qianfoshan Hospital. Firstly, the total RNA of samples was isolated and purified by TRIzol reagent following the instruction manual. The concentration of RNA was quantified by NanoPhotometer N50. Then, reverse transcription of mRNA was carried out using the SureScript-First-strand-cDNA-synthesis-kit (Servicebio, China). Next, the reverse transcription product cDNA was used as template to run 40 cycles of reaction, including 1 min at 95° C (pre-denaturation), followed by at 95° C for 20 s (denaturation), 55° C for 20 s (annealing) and 72° C for 30 s (elongation) on the CFX96 real-time quantitative PCR instrument. Primer sequences were shown in Table 1.

**Table 1:** Primers used for qPCR.

**Gene name**	**Forward primer sequence**	**Reverse primer sequence**
ACTN1	CCTCCCTGATGCCGACAAG	CCTGAGGCGTGATGGTTGT
F5	TAAGCCCTTGAGCATCCATCC	TGGGTCCACTGTCCTCACTGATA
JMJD1C	CCAACTCTAATACCCGAACCAA	CCATAGCAGCCTGTAACTTTCC
TLN1	CGTGCAAACCAGGCAATTCA	ATTGGTGGTACGGGCAGAAG
WAS	GCGACTCTTTGAGATGCTTGG	CGTAAAGGCGGATGAAGTAGGA
GAPDH	CGAAGGTGGAGTCAACGGATTT	ATGGGTGGAATCATATTGGAAC

### Data availability statement

The datasets GSE16561 and GSE58294 for this study can be downloaded in the GEO database (https://www.ncbi.nlm.nih.gov/geo/). The processed data are available from the corresponding author upon reasonable request.

## Supplementary Material

Supplementary Figure 1

Supplementary Table 1

Supplementary Table 2
