# Supplementary figures and images for: Machine learning identifies novel coagulation genes as diagnostic and immunological biomarkers in ischemic stroke
Source: Aging (Albany NY). 2024 Apr 3;16(7):6314–33. doi: 10.18632/aging.205706 (PMC11042924; doi:10.18632/aging.205706)

## SUPPLEMENTARY FIGURE

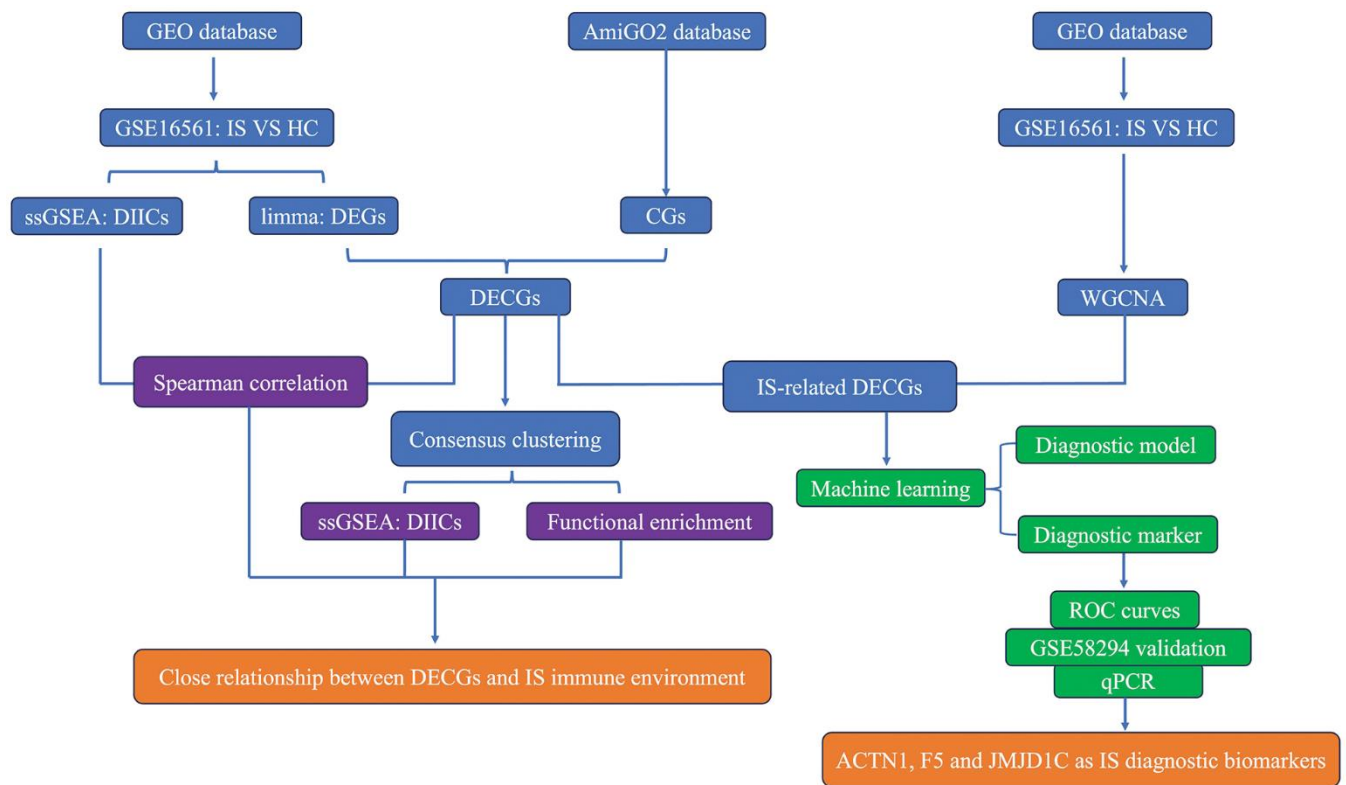

**Supplementary Figure 1. The schematic workflow of the current study.**

Supplement: Supplementary Figure 1 [file aging-16-205706-s001.pdf]
